# Annotations

**Published:** 1907-07-27

**Authors:** 


### Annotations.

### Heart Disease and Pregnancy.

The suggestion raised by the conjunction of these two terms is one which has an urgent and anxious aspect for the medical practitioner. Very different opinions have been expressed by medical writers as to the effect likely to be produced by pregnancy on patients suffering from valvular disease. The late Dr. Angus Macdonald, in his book dealing with this subject, held a very gloomy outlook, and this more especially in reference to the victims of mitral stenosis, which, of course, is one of the commonest affections in young women. Some 18 years ago Dr. Macdonald's contention was subjected to a critical examination by Dr. Middleton, of Glasgow, who recorded a number of cases observed in hospital practice and watched over a period of years. Dr. Middleton's conclusions were of a much more favourable nature, and, writing of mitral stenosis, he stated that even when pregnancy occurs the prognosis must be regarded "with considerable hopefulness." A somewhat similar presentation of the discussion, based upon the obstetrical histories of 300 cases, has more recently been published by Dr. Hicks and Dr. French. Now Dr. G. F. Blacker adds his verdict in the same direction. There are risks, he admits, but the risks have been greatly exaggerated. He doubts whether the inevitable consequences of valvular disease are appreciably hastened by child-bearing—at all events they are not so certainly hastened as to justify a refusal of the right to marry. With this statement there must be coupled the assumption that the valvular flaw is compensated, and that there is no disease of the lungs or other viscera. When all the above authorities are considered, it is seen that the older view, according to which cardiac disease in a woman was an absolute bar to marriage, must be very decidedly modified.

### Syphilis and the State.

In the recent discussion on this subject at the Royal Academy of Medicine in Ireland, some of the contributions included references to the widespread effects of syphilis both in the army and navy and in civil life, and to the economic losses thus inflicted on the nation. Dr. Seymour Stritch estimated that the immediate cost to the nation of venereal diseases in the public services is not less than £3,000,000 annually, and if indirect consequences were considered, such, for example, as superannuations, and sick leave, the charge must be raised to something like £7,000,000. He considers that the Contagious Diseases Acts exercise a favourable influence, and advises their application, not merely to limited and restricted areas, but to all parts of the Empire. He also suggested that a special course of study in dermatology and syphilis should be included in the medical curriculum, and that lectures on the dangers of venereal diseases should be given in schools and in ambulance classes. Dr. Pugin Meldon agreed that the absence of legislative measures in regard to syphilitic patients makes it difficult to check the spread of the disease. Possibly, the economical aspect of the question as presented by Dr. Stritch may impress public opinion, but until the suspicion that legislative measures would promote prostitution is dissipated, it is hopeless to expect the interference of Parliament. Several speakers questioned the advisability of compulsory measures on the part of the State, and pointed out that any such proceedings would offend the moral sense of a large section of the community. It may also be questioned whether such a policy, even allowing it to be advisable, can be successfully applied in practice. The morbidity and mortality of venereal diseases are certainly serious elements in the chapter of national well-being.

### The Medical Inspection of School Children.

Though other aspects of elementary education have proved, only too often, a fruitful source of long and bitter controversy, all parties are agreed that the national interests demand an ordered and systematic application of medical knowledge to the supervision and control of the rising generation. It may indeed be taken as certain that any future Bill that may be presented to Parliament as a national educational scheme will contain a clause to secure this end. Legislation, however, will be of but small avail unless it provides for responsible and effective administration. Hence we are in entire sympathy with the writer of a recent article in the *Times* opposing the suggestion that the medical inspection of schools should be regarded merely as a part of the general sanitary administration of the country, and should be placed in the charge of the medical department of the Local Government Board. This would mean little more than the application to the schools of the general sanitary and hygienic methods which it is the duty of this Board to apply in various other directions. What is wanted is something much more definitely specialised and much more immediately in contact with the essential processes of the scheme of elementary education. The medical control needed must be applied not merely to the school buildings, but especially and particularly to the scholars. It must deal, in association with the teacher, with the problems which arise in connection with intellectual deficiency, with underfeeding, and with all those abnormal diseased conditions of the children by which the educational progress of the scholar is hindered. Beyond these pressing claims it is much to be desired that statistics shall be collected for the purpose of organising a regular anthropometrical survey, which can hardly fail to prove of great interest and importance. It is, we agree, not to be expected that these ends can be attained unless medical expert opinion has an opportunity for full expression at the Board of Education itself, and we therefore cordially support the suggestion that a medical department should be constituted at that Board so that the responsible Minister may be advised on all the medical aspects of educational questions.

